# Segmental bioimpedance in pregnant end stage renal failure patient for dry weight titration and volume management (case report)

**DOI:** 10.1186/s12882-023-03360-6

**Published:** 2023-10-24

**Authors:** Sabrina Haroon, Jia Neng Tan, Titus Lau, Shiao-Yng Chan, Andrew Davenport

**Affiliations:** 1 Division of Nephrology, University Medicine Cluster, National University Hospital Singapore, Level 10, NUHS Tower Block, 1E Kent Ridge Road, 119228 Singapore, Republic of Singapore; 2 Department of Obstetrics and Gynecology, School of Medicine, National University Hospital and Yong Loo Lin, National University of Singapore, Singapore, Republic of Singapore; 3 Department of Renal Medicine, University College London, Royal Free Hospital, London, UK

**Keywords:** Pregnancy, Segmental bioimpedance, Haemodialysis

## Abstract

**Background:**

Volume assessment, dry weight titration, and blood pressure control in pregnant kidney failure patients are often challenging, with physiological fluid accumulation in the trunk and lower limbs and an increased risk of preeclampsia. We used segmental bioimpedance in the volume management of our kidney failure patient on haemodialysis.

**Case presentation:**

We report a case of a female patient on maintenance haemodiafiltration with no residual kidney function for whom we used segmental bioimpedance to guide dry weight adjustment. At different gestational periods, we targeted a different extracellular to total body water ratio according to body segments. This allowed us to support her high-risk pregnancy, identify her as probably developing preeclampsia and trigger a plan for closer monitoring and delivery during the third trimester when she had rapid weight gain.

**Conclusion:**

Segmental bioimpedance is a practical, simple, and non-invasive test that can be performed at the dialysis unit and is useful as an adjunct decision-making tool in the management of pregnant dialysis patients.

**Supplementary Information:**

The online version contains supplementary material available at 10.1186/s12882-023-03360-6.

## Background

Pregnancy is not only uncommon in patients on maintenance dialysis without residual renal function but is associated with high maternal and foetal morbidity and mortality [1, 2]. Target weight must be titrated weekly during pregnancy, assuming healthy foetal growth. However, volume assessment in pregnancy is often challenging with physiological fluid accumulation in the trunk and lower limbs. Volume management to avoid hypervolemia and hypovolemia is crucial to prevent haemodynamic placental stress while ensuring cardiovascular stability on dialysis.

We report a female anuric haemodialysis (HD) patient who conceived naturally. After confirming pregnancy, she was converted from HD to haemodiafiltration (HDF) to improve overall solute clearances and ensure haemodynamic stability. Post-dialysis segmental bioimpedance was used to guide the clinical assessment of volume status. Our aim was to prevent fluctuations in blood pressure (BP), hypovolemia, intradialytic hypotension and hypertension and to preserve placenta perfusion. We postulated that optimal volume status with accurate titration of target weight using segmental bioimpedance, in addition to good clinical management, would allow better BP control and enable pregnancy extension to term. We used a total body and upper limb extracellular water to total body water ratio (ECW/TBW) for dry weight titration during her second and third trimester, respectively.

## Case presentation

A 25-year-old lady who had been HD dependent for 4 years due to IgA nephropathy was confirmed pregnant in 2022. Her medical history included hypertension on amlodipine, hyperparathyroidism with post-subtotal parathyroidectomy, and childhood asthma. Pre-pregnancy, her body mass index (BMI) was normal at 20.9 kg/m2 [3]. An initial ultrasound scan confirmed viable singleton intrauterine pregnancy at 9 weeks gestation. She was switched to pre-dilution HDF six-hour sessions, six times a week for improved solute clearance. Given that blood flow to the placenta is not autoregulated and can fall during dialysis sessions, HDF was preferred because of more stable haemodynamics from possibly more significant thermal losses [4]. Her vascular access was with a left brachio-cephalic fistula, and HDF sessions used a blood flow (Qb) of 250 mL/min with ultrapure dialysate at 500 mL/min flow rate. As pregnancy is pro-thrombotic and her blood flow rate was lower than previous published reports on HDF in pregnancy, we have chosen pre-dilution HDF to achieve a higher convective volume and to reduce extracorporeal circuit clotting [5]. In addition, we chose pre-dilution HDF as studies comparing high volume HDF in pre-dilution and post-dilution mode, using comparable convection volume exchange rates, have shown that pre-dilution mode has reduced sieving coefficients for sodium, calcium and magnesium, and thus losses of calcium and magnesium would be expected to be less with pre-dilution compared to post-dilution mode [6]. The convective volume was fixed at 55.0 L/session, keeping the pre-dialysis serum urea concentration < 12.5 mmol/L [7]. The dialysate composition comprised 31 mmol/L bicarbonate, 1.5 mmol/L calcium, 3.0 mmol/L potassium, and 136 mmol/L sodium, with a dialysate temperature of 35.5 °C. Anticoagulation was with unfractionated heparin.

BP was controlled with regular long-acting Nifedipine 60 mg twice daily and Methyldopa 250 mg daily. The erythropoietin dose was increased to achieve a target haemoglobin of 100 g/L. Her baseline transferrin saturation and ferritin were 56.5% and 738 ug/L, respectively. She was allowed an unrestricted, high protein diet supplemented with folic acid, and prenatal vitamins [8]. Our patient did not require any potassium or magnesium supplementation.

Weekly segmental bioimpedance was measured with the InBody S10 (Seoul, South Korea) in a standardized manner 20 min after completing the dialysis session, after allowing time for re-equilibration and blood test results were checked to ensure optimum volume management and guide dialysis target weight setting throughout pregnancy (Online supplementary material). Pre-dialysis measurements differ from post-dialysis measurements, as patients are usually volume overloaded in the pre-dialysis state hence, bioimpedance measurements overestimate muscle mass and under estimate fat mass, due to the difference in the water content between muscle (≈ 90% water and fat tissue < 10%) [9].

**Fig. 1:**
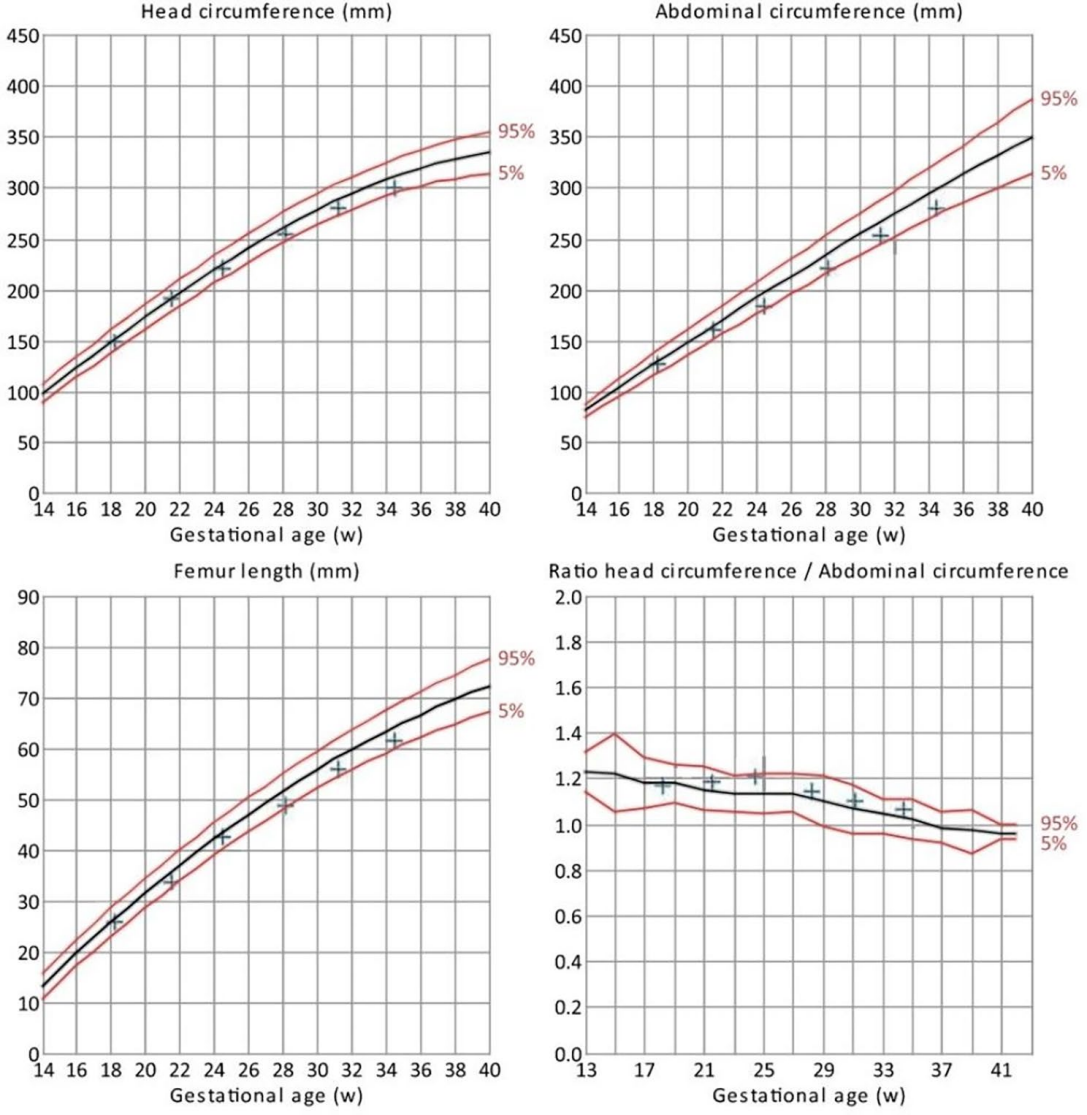
Fetal growth during pregnancy

An ultrasound scan at 18 weeks demonstrated an absence of the right umbilical artery, with a further scan at 21 weeks demonstrating bilateral increased uterine artery resistance with unilateral notching of the right uterine artery Doppler. Her dialysis sessions were lengthened to 6.5 hour, Qb was increased to 270 mL/min, and convective volume to 60 L at 20 weeks. With this change, she remained well with good BP control. The standard weekly Kt/V measured 6.71 to 7.5. At 21 weeks, she tested positive for COVID-19 infection, and was hospitalized for one week. She lost weight during her hospitalization and was not able to tolerate oral nutritional supplementation. We observed a reduction in total body ECW/TBW post COVID-19 admission from 0.391 to 22 weeks gestation to 0.379 at 26 weeks gestation (Fig. 1). During the same period, we observed a fall in body fat mass which subsequently increased post hospital discharge (Online supplementary material). The findings from bioimpedance were consistent with a higher weight gain from her improved appetite and oral intake post infection. Using bioimpedance targets and serial measurements, we increased her dry weight by 0.9–1.7 kg per week, much higher compared to what would normally be anticipated during the second trimester pregnancy [10]. Her weight change in the second trimester ranged from − 0.2 to 0.7 kg/ week, with a total weight gain of 2.9 kg at the end of 26 weeks.

From 30 weeks gestation, her pregnancy was subsequently complicated by labile BP with intradialytic systolic BPs up to 200 mmHg, occasionally requiring additional antihypertensive medication during dialysis. In the third trimester, with the physiological changes, we observed a higher ECW/TBW in trunk (0.383–0.402) and lower limb (0.384–0.409) compared to non-access upper limb (0.372–0.389), as expected. Her body fat mass increased during this period from 18.9 to 23.3 kg while skeletal muscle mass remained stable. Her weight gain ranged from 0.7 to 3.2 kg/ week, with a total weight gain of 12.4 kg. Data from serial bioimpedance measurements provided additional information to account for the increasing body weight, differentiating it from overhydration. Her intradialytic hypertension improved each time with up-titration of target weight guided by bioimpedance but there was also a suspicion that this could represent early signs of pre-eclampsia. Foetal size was below average, but growth velocity was sustained throughout the third trimester (Fig. 1). She started experiencing irregular tightenings at 34 weeks^+ 3 days^ and upon admission was found to have an escalating BP with shortness of breath and pulmonary oedema clinically, consistent with clinical features of rapidly developing pre-eclampsia, hence, a caesarean section was performed. The baby was healthy, weighing 1.9 kg with an Apgar Score of 9 at 1 min.

## Discussion and conclusions

**Fig. 2:**
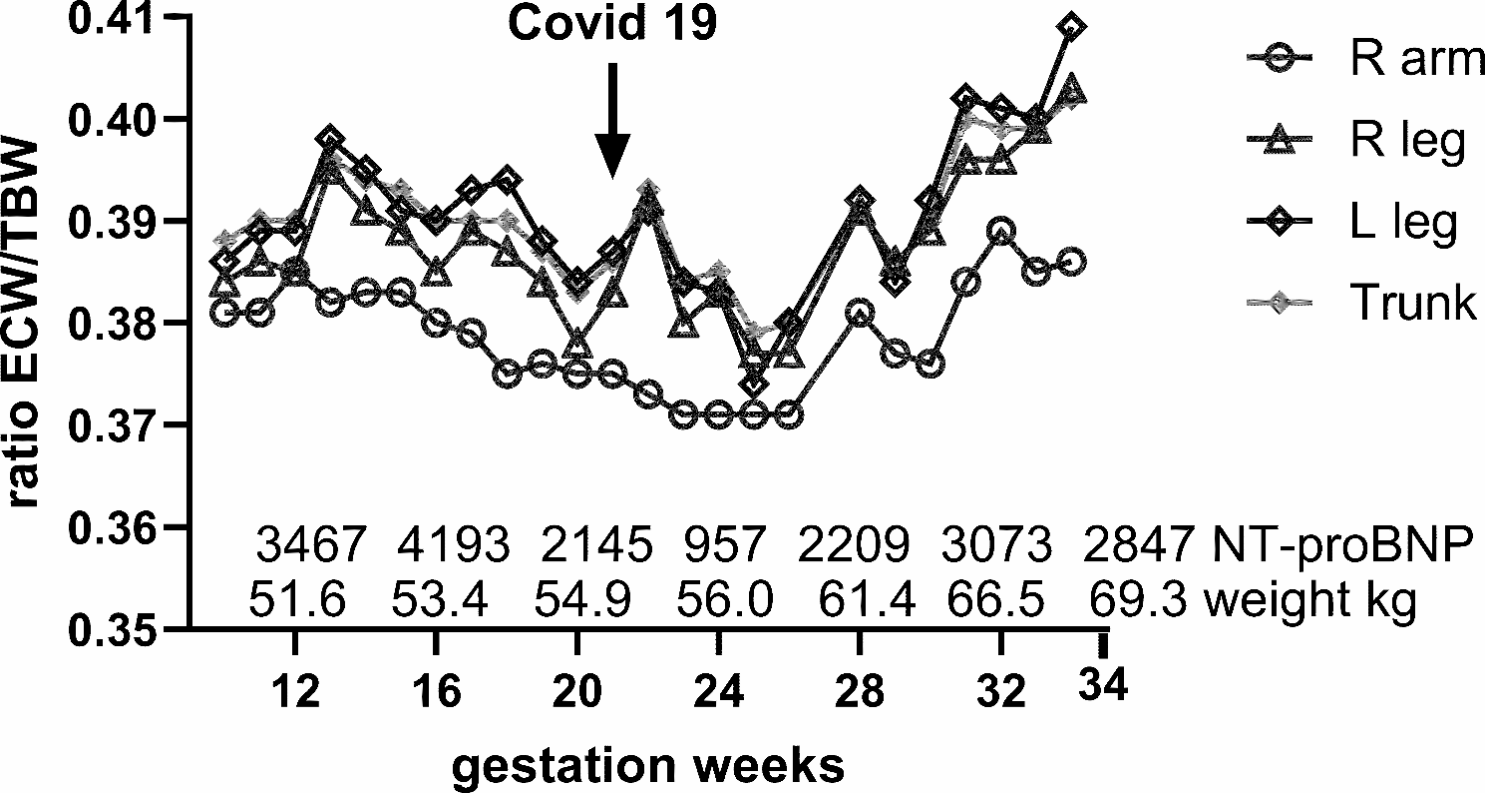
ECW/TBW ratio over pregnancy in in different body segments

We present a case of a maintenance dialysis patient who successfully continued her pregnancy to 34 weeks and delivered a healthy baby. Although she lost weight during her second trimester due to nausea and COVID-19 infection, this was followed by more ideal weight gains in the third trimester. As BP is closely related to volume status in kidney failure patients, we used segmental bioimpedance during her pregnancy to guide dialysis weight targets and volume assessment. In the second trimester, dry weight titration was guided by total body ECW/TBW using 0.39 as a target. To allow for the physiological changes during the third trimester, we used ECW/TBW of 0.39 at upper limbs, specifically in the right arm without vascular access, while allowing a higher ECW/TBW in the trunk and lower limbs to account for the growing foetus, accumulating amniotic fluid and peripheral oedema in the lower limbs (Fig. 2).

The Institute of Medicine recommends a total gestational weight gain of 11-16 kg for a healthy woman starting with a normal BMI [10]. Our patient gained 16 kg in her pregnancy from a pre-weight of 52 kg. While healthy gestational weight gain is estimated to be 300 g/week during the second trimester and 300–500 g/week during the third trimester, in reality, this is less predictable in a maintenance dialysis patient, especially with an intercurrent acute illness [11]. Weight allowance must be carefully titrated. Our patient had lower than expected weight gain in her second trimester but gained weight rapidly in the third. We believe the weight gain in the third trimester is related to catching up from the weight loss from the second trimester, improved appetite with no more nausea, related to a more restricted lifestyle during the pandemic, as well as to third spacing associated with developing preeclampsia [12, 13]. Her total weight gain was 16 kg, which is towards the upper limit of that recommended for her BMI [10].

Our patient had established hypertension pre-pregnancy, and BP was controlled with Nifedipine LA and Methyldopa during her pregnancy. We avoided beta-blockers with her history of asthma. Preeclampsia affects up to one-third of women with chronic kidney disease (CKD) and is thought to be driven by a placental disorder that leads to maternal endothelial dysfunction. While biomarkers, including soluble fms-like tyrosine kinase (sFlt-1) and placenta growth factor, may aid in predicting preeclampsia, these tests are not readily available and have a lower predictive performance in kidney failure patients [14]. Also, the diagnosis is often challenging in CKD as serum creatinine is elevated at baseline, residual renal function is variable and diagnostic criteria of proteinuria cannot be evaluated.

Cross-sectional studies have compared bioimpedance measurements of ECW and ICW with those of gold standard isotope dilution methods, and bioimpedance spectroscopy was not found to be superior to single frequency (50 Hz) measurements in dialysis patients [15]. We used a multifrequency bioimpedance device that could measure ICW and ECW in body segments rather than whole body measurements and provided information on nutritional status [16, 17]. While bioimpedance has proven useful in dialysis patients to guide target weight adjustments, it has not been shown to accurately measure changes in ECW/TBW during dialysis or prevent intra-dialytic hypotension [18–20]. Therefore, we used a protocol based on studies that had investigated the time required for re-equilibration of fluid between body compartments post-dialysis and the time required to obtain stable bioimpedance measurements [20]. By making serial measurements in the same patient and using ratios of body fluid compartments, we minimised any systematic errors in bioimpedance measurements.

Segmental bioimpedance allowed a more accurate adjustment of the dry target, given the accumulation of amniotic fluid in the trunk and fluid retention in the legs with advancing gestational age. Specific to pregnancy, bioimpedance has been reported to be able to distinguish patients developing preeclampsia from those having merely pregnancy-induced hypertension and to identify those at risk of having small for gestational age foetuses at an earlier gestational stage [21]. Bioimpedance in pregnant patients also showed good accuracy and high negative predictive value for the risk of developing preeclampsia [22].

Pregnant dialysis patients are at increased risk of developing preeclampsia. Its diagnosis and the decision and timing of delivery are often challenging, striking a balance between the risk of worsening preeclampsia and its associated morbidity against the risk of foetal prematurity. Segmental bioimpedance in our patient showed rapid weight gain from 28 weeks onwards and provided us with information that was important in our discussion with the obstetric team on the anticipated timing of delivery and administration of dexamethasone. Segmental bioimpedance is a simple, fast, and non-invasive test that can help better support pregnancy and identify high risk patients.

### Electronic supplementary material

Below is the link to the electronic supplementary material.

Supplementary Material 1

Supplementary Material 2

## Data Availability

All data used for the case report are included in this published article and its supplementary information files.

## List of abbreviations

HD: haemodialysis
HDF: hemodiafiltration
BP: blood pressure
BMI: body mass index
Qb: blood flow (Qb)
ECW/TBW: extracellular water to total body water ratio
CKD: chronic kidney disease
sFlt-1: soluble fms-like tyrosine kinase
BIA: bioimpedance analysis
BIS: bioimpedance spectroscopy

## References

[1] Bagon JA, Vernaeve H, De Muylder X, Lafontaine JJ, Martens J, Van Roost G (1998). Pregnancy and dialysis. Am J Kidney Dis.

[2] Successful pregnancies in women treated by dialysis and kidney transplantation (1980). Report from the Registration Committee of the european Dialysis and Transplant Association. Br J Obstet Gynaecol.

[3] American College of O, Gynecologists (2013). ACOG Committee opinion no. 548: weight gain during pregnancy. Obstet Gynecol.

[4] Locatelli F, Altieri P, Andrulli S, Bolasco P, Sau G, Pedrini LA (2010). Hemofiltration and hemodiafiltration reduce intradialytic hypotension in ESRD. J Am Soc Nephrol.

[5] Puddu M, Migueliz ML, Rosa-Diez G, Crucelegui S, Martinez AH, Luxardo R (2021). High volume on line hemodiafiltration in dialysis pregnant patients: predilutional or postdilutional?. J Artif Organs.

[6] Masakane I, Kikuchi K, Kawanishi H (2017). Evidence for the clinical advantages of Predilution On-Line hemodiafiltration. Contrib Nephrol.

[7] Wiles K, Chappell L, Clark K, Elman L, Hall M, Lightstone L (2019). Clinical practice guideline on pregnancy and renal disease. BMC Nephrol.

[8] 21st Century. :21st CenturyPrenatalVitamins https://www.21stcenturyvitamins.com/products/multivitamins/prenatal. Accessed 25 Aug 2023.

[9] Tangvoraphonkchai K, Davenport A (2017). Changes in body composition following haemodialysis as assessed by bioimpedance spectroscopy. Eur J Clin Nutr.

[10] Rasmussen KM, Yaktine AL, editors. Weight Gain During Pregnancy: Reexamining the Guidelines. The National Academies Collection: Reports funded by National Institutes of Health. Washington (DC)2009. 20669500

[11] Cabiddu G, Castellino S, Gernone G, Santoro D, Giacchino F, Credendino O (2015). Best practices on pregnancy on dialysis: the italian study group on kidney and pregnancy. J Nephrol.

[12] Carfi A, Bernabei R, Landi F, Gemelli Against C-P-ACSG (2020). Persistent symptoms in patients after Acute COVID-19. JAMA.

[13] Bhutani S, vanDellen MR, Cooper JA. Longitudinal Weight Gain and related risk behaviors during the COVID-19 pandemic in adults in the US. Nutrients. 2021;13(2). 10.3390/nu13020671 PMC7922943 33669622

[14] Wiles K, Bramham K, Seed PT, Brockbank A, Nelson-Piercy C, Karumanchi SA (2021). Placental and endothelial biomarkers for the prediction of superimposed pre-eclampsia in chronic kidney disease. Pregnancy Hypertens.

[15] Raimann JG, Zhu F, Wang J, Thijssen S, Kuhlmann MK, Kotanko P (2014). Comparison of fluid volume estimates in chronic hemodialysis patients by bioimpedance, direct isotopic, and dilution methods. Kidney Int.

[16] Booth J, Pinney J, Davenport A (2011). The effect of vascular access modality on changes in fluid content in the arms as determined by multifrequency bioimpedance. Nephrol Dial Transplant.

[17] Davenport A (2013). Does peritoneal dialysate affect body composition assessments using multi-frequency bioimpedance in peritoneal dialysis patients?. Eur J Clin Nutr.

[18] Davies SJ, Davenport A (2014). The role of bioimpedance and biomarkers in helping to aid clinical decision-making of volume assessments in dialysis patients. Kidney Int.

[19] Haroon S, Tai BC, Yeo X, Davenport A (2022). Changes in total and segmental extracellular and intracellular volumes with hypotension during hemodialysis measured with bioimpedance spectroscopy. Artif Organs.

[20] Tangvoraphonkchai K, Davenport A (2016). Do Bioimpedance measurements of Over-Hydration accurately reflect Post-Haemodialysis Weight. Changes? Nephron.

[21] Piuri G, Ferrazzi E, Bulfoni C, Mastricci L, Di Martino D, Speciani AF (2017). Longitudinal changes and correlations of bioimpedance and anthropometric measurements in pregnancy: simple possible bed-side tools to assess pregnancy evolution. J Matern Fetal Neonatal Med.

[22] Trindade CR, Torloni MR, Mattar R, Sun SY (2021). Good performance of bioimpedance in early pregnancy to predict preeclampsia. Pregnancy Hypertens.

