# Supplementary material for: Segmental bioimpedance in pregnant end stage renal failure patient for dry weight titration and volume management (case report)
Source: BMC Nephrol. 2023 Oct 24;24:308. doi: 10.1186/s12882-023-03360-6 (PMC10598990; doi:10.1186/s12882-023-03360-6)
Supplement: Supplementary file 1 — Supplementary Material 1 [file 12882_2023_3360_MOESM1_ESM.docx]

**Weight changes and segmental bioimpedance information according to pregnancy week**

| Gestation weeks | | Pre- HD Weight (kg) | Post-HD Weight (kg) | Dry  Weight  (kg) | NT-BNP | Weight at bioimpedance measurement  (kg) | Extracellular water/ Total body water (ECW/TBW) | | | | Body Fat Mass (kg) | Weight change (kg) |
| --- | --- | --- | --- | --- | --- | --- | --- | --- | --- | --- | --- | --- |
|  |  |  |  |  |  |  | Total | Trunk | UL | LL | Skeletal Muscle Mass (kg) |  |
| 16/8/22 (10  weeks) | | 53.5 | 51.7 | 51.3 | 6468 | 51.7 | 0.387 | 0.388 | Right 0.381 | Right 0.384 | 10.2 |  |
|  |  |  |  |  |  |  |  |  | Left 0.387 | Left 0.386 | 22.2 |  |
| 23/8/22 (11 weeks) | | 52.9 | 51.4 | 51.6 | 5025 | 51.4 | 0.388 | 0.390 | Right 0.381 | Right 0.386 | 10.3 | 0.3 |
|  |  |  |  |  |  |  |  |  | Left 0.384 | Left 0.389 | 21.9 |  |
| 30/8/22 (12 weeks) | | 52.6 | 51.6 | 51.8 | 3467 | 51.6 | 0.389 | 0.390 | Right 0.385 | Right 0.385 | 11 | 0.2 |
|  |  |  |  |  |  |  |  |  | Left 0.390 | Left 0.389 | 21.5 |  |
| 7/9/22  (13 weeks) | | 53.4 | 52.4 | 52.5 | 4319 | 52.4 | 0.395 | 0.396 | Right 0.382 | Right 0.395 | 11.9 | 0.7 |
|  |  |  |  |  |  |  |  |  | Left 0.386 | Left 0.398 | 21.4 |  |
| 14/9/22 (14 weeks) | | 54 | 52.7 | 52.7 | 3614 | 52.7 | 0.392 | 0.394 | Right 0.383 | Right 0.391 | 12.4 | 0.2 |
|  |  |  |  |  |  |  |  |  | Left 0.386 | Left 0.395 | 21.3 |  |
| 21/9/22 (15 weeks) | | 54.3 | 52.8 | 52.9 | 3385 | 52.8 | 0.391 | 0.393 | Right 0.383 | Right 0.389 | 13.6 | 0.2 |
|  |  |  |  |  |  |  |  |  | Left 0.388 | Left 0.391 | 20.7 |  |
| 28/9/22 (16 weeks) | | 54.3 | 53.2 | 53.4 | 4193 | 53.2 | 0.388 | 0.39 | Right 0.380 | Right 0.385 | 12.8 | 0.5 |
|  |  |  |  |  |  |  |  |  | Left 0.386 | Left 0.390 | 21.4 |  |
| 5/10/22 (17 weeks) | | 54.9 | 53.6 | 53.7 | 4503 | 53.6 | 0.389 | 0.39 | Right 0.379 | Right 0.389 | 12.8 | 0.3 |
|  |  |  |  |  |  |  |  |  | Left 0.388 | Left 0.393 | 21.7 |  |
| 12/10/22 (18 weeks) | | 54.6 | 53.8 | 53.9 | 3772 | 53.8 | 0.388 | 0.39 | Right 0.375 | Right 0.387 | 12.7 | 0.2 |
|  |  |  |  |  |  |  |  |  | Left 0.377 | Left 0.394 | 21.9 |  |
|  |  | | | | | | | | | | | |
| 19/10/22 (19 weeks) | | 55.7 | 54.3 | 54.4 | 2631 | 54.3 | 0.385 | 0.387 | Right 0.376 | Right 0.384 | 13.5 | 0.5 |
|  |  |  |  |  |  |  |  |  | Left 0.379 | Left 0.388 | 21.8 |  |
| 26/10/22 (20 weeks) | | 56.2 | 54.9 | 55 | 2145 | 54.9 | 0.382 | 0.383 | Right 0.375 | Right 0.378 | 15.2 | 0.6 |
|  |  |  |  |  |  |  |  |  | Left 0.383 | Left 0.384 | 21.3 |  |
| 2/11/22 (21 weeks) | | 56.8 | 55.9 | 55.7 | 1648 | 55.9 | 0.385 | 0.386 | Right 0.375 | Right 0.383 | 16.4 | 0.7 |
|  |  |  |  |  |  |  |  |  | Left 0.380 | Left 0.387 | 21.1 |  |
|  | Admitted 6/11/22 – 11/11/22 (COVID) | | | | | | | | | | | |
| 12/11/22 (22 weeks) | | 56.9 | 55.3 | 55.5 | NA | 55.3 | 0.391 | 0.393 | Right 0.373 | Right 0.392 | 15.8 | -0.2 |
|  |  |  |  |  |  |  |  |  | Left 0.377 | Left 0.391 | 20.9 |  |
| 16/11/22 (23 weeks) | | 57.3 | 55.6 | 55.5 | 1171 | 55.6 | 0.382 | 0.384 | Right 0.371 | Right 0.380 | 14.1 | 0 |
|  |  |  |  |  |  |  |  |  | Left 0.377 | Left 0.384 | 22.3 |  |
| 23/11/22 (24 weeks) | | 57.2 | 56 | 55.6 | 957 | 56 | 0.383 | 0.385 | Right 0.371 | Right 0.383 | 15.4 | 0.1 |
|  |  |  |  |  |  |  |  |  | Left 0.373 | Left 0.383 | 21.8 |  |
| 30/11/22 (25 weeks) | | 58.2 | 56.9 | 56.5 | 973 | 56.9 | 0.377 | 0.379 | Right 0.371 | Right 0.377 | 16.1 | 0.9 |
|  |  |  |  |  |  |  |  |  | Left 0.372 | Left 0.374 | 22.0 |  |
| 7/12/22 (26 weeks) | | 59.7 | 58.1 | 58.2 | 1004 | 58.1 | 0.379 | 0.38 | Right 0.371 | Right 0.377 | 16 | 1.7 |
|  |  |  |  |  |  |  |  |  | Left 0.376 | Left 0.380 | 22.7 |  |
| 14/12/22  (27 weeks) | | 61.2 | 59.7 | 59.9 | 1573 | 59.7 | 0.381 | 0.383 | Right 0.372 | Right 0.382 | 16.2 | 1.7 |
|  |  |  |  |  |  |  |  |  | Left 0.377 | Left 0.379 | 23.5 |  |
| 21/12/22 (28 weeks) | | 63 | 61.5 | 61.4 | 2209 | 61.5 | 0.39 | 0.391 | Right 0.381 | Right 0.391 | 18.9 | 1.5 |
|  |  |  |  |  |  |  |  |  | Left 0.384 | Left 0.392 | 22.7 |  |
| 28/12/22 (29 weeks) | | 63.9 | 62.3 | 62.2 | 1658 | 62.3 | 0.384 | 0.385 | Right 0.377 | Right 0.386 | 19.5 | 0.8 |
|  |  |  |  |  |  |  |  |  | Left 0.380 | Left 0.384 | 23.0 |  |
| 04/01/23 (30 weeks) | | 64.4 | 63.9 | 63.8 | 1577 | 63.9 | 0.388 | 0.39 | Right 0.376 | Right 0.389 | 19.8 | 1.6 |
|  |  |  |  |  |  |  |  |  | Left 0.378 | Left 0.392 | 23.6 |  |
| 11/01/23 (31 weeks) | | 66.3 | 65.8 | 65.2 | 2458 | 65.8 | 0.398 | 0.4 | Right 0.384 | Right 0.396 | 21.8 | 1.4 |
|  |  |  |  |  |  |  |  |  | Left 0.389 | Left 0.402 | 23.2 |  |
| 18/01/23 (32 weeks) | | 67.2 | 66.5 | 66 | 3073 | 66.5 | 0.398 | 0.399 | Right 0.389 | Right 0.396 | 22.4 | 0.8 |
|  |  |  |  |  |  |  |  |  | Left 0.392 | Left 0.401 | 23.3 |  |
| 24/01/23 (33 weeks) | | 67.7 | 67 | 67.3 | 1192 | 67 | 0.398 | 0.399 | Right 0.385 | Right 0.399 | 23.8 | 1.3 |
|  |  |  |  |  |  |  |  |  | Left 0.390 | Left 0.400 | 22.8 |  |
| 30/01/23 (34 weeks) | | 71 | 69.3 | 68 | 2847 (1/2/23) | 69.3 | 0.402 | 0.402 | Right 0.386 | Right 0.403 | 23.3 | 0.7 |
|  |  |  |  |  |  |  |  |  | Left 0.392 | Left 0.409 | 24.2 |  |
