# Supplementary material for: Segmental bioimpedance in pregnant end stage renal failure patient for dry weight titration and volume management (case report)
Source: BMC Nephrol. 2023 Oct 24;24:308. doi: 10.1186/s12882-023-03360-6 (PMC10598990; doi:10.1186/s12882-023-03360-6)
Supplement: Supplementary file 2 — Supplementary Material 2 [file 12882_2023_3360_MOESM2_ESM.docx]

**Laboratory test according to pregnancy week**

| Gestation weeks | Urea  (mmol/L) | Creatinine (umol/L) | Sodium  (mmol/L) | Potassium (mmol/L) | Calcium (mmol/L) | Albumin  (g/L) | Phosphate (mmol/L) | Magnesium (mmol/L) |
| --- | --- | --- | --- | --- | --- | --- | --- | --- |
| 16/8/22 (10 weeks) | 8.4 | 338 | 138 | 3.8 | 2.19 | 35 | 0.94 | NIL |
| 23/8/22 (11 weeks) | 7.8 | 320 | 137 | 3.6 | 2.29 | 37 | 0.85 | NIL |
| 30/8/22 (12 weeks) | 5.8 | 347 | 137 | 3.7 | 2.30 | 35 | 0.82 | NIL |
| 7/9/22 (13 weeks) | 9.6 | 400 | 135 | 4.3 | 2.10 | 32 | 0.50 | 0.80 |
| 14/9/22 (14 weeks) | 10.8 | 433 | 135 | 4.5 | 2.07 | 35 | 0.95 | 0.82 |
| 21/9/22 (15 weeks) | 11.7 | 447 | 137 | 4.2 | 2.03 | 33 | 0.94 | 0.75 |
| 28/9/22 (16 weeks) | 10.7 | 435 | 136 | 3.8 | 2.03 | 33 | 0.75 | 0.76 |
| 5/10/22 (17 weeks) | 13.7 | 430 | 133 | 4.3 | 2.05 | 33 | 0.80 | 0.76 |
| 12/10/22 (18 weeks) | 11.1 | 426 | 136 | 4.2 | 2.10 | 34 | 1.15 | 0.82 |
| 19/10/22 (19 weeks) | 14.8 | 440 | 137 | 4.1 | 2.09 | 35 | 0.84 | 0.77 |
| 26/10/22 (20 weeks) | 16.8 | 456 | 138 | 4.3 | 2.06 | 34 | 0.99 | 0.82 |
| 2/11/22 (21 weeks) | 10.0 | 400 | 132 | 3.9 | 2.07 | 34 | 0.95 | NIL |
| Admitted 6/11/22 – 11/11/22 (COVID) | | | | | | | | |
| 12/11/22 (22 weeks) | 9.0 | 497 | 135 | 3.9 | NA | 31 | NA | NA |
| 16/11/22 (23 weeks) | 12.5 | 406 | 134 | 3.9 | 2.08 | 35 | 1.12 | 0.81 |
| 23/11/22 (24 weeks) | 13.8 | 443 | 136 | 4.5 | 2.08 | 36 | 1.29 | 0.80 |
| 30/11/22 (25 weeks) | 11.8 | 427 | 134 | 4.4 | 2.16 | 34 | 1.01 | 0.85 |
| 7/12/22 (26 weeks) | 12.4 | 409 | 135 | 4.2 | 2.02 | 33 | 1.05 | 0.79 |
| 14/12/22 (27 weeks) | 14.0 | 402 | 135 | 4.1 | 2.01 | 33 | 1.50 | 0.76 |
| 21/12/22 (28 weeks) | 11.2 | 436 | 134 | 4.3 | 2.09 | 35 | 0.98 | 0.76 |
| 28/12/22 (29 weeks) | 13.4 | 417 | 134 | 4.3 | 1.99 | 32 | 1.03 | 0.80 |
| 04/01/23 (30 weeks) | 10.5 | 382 | 132 | 4.3 | 2.08 | 33 | 1.16 | 0.82 |
| 11/01/23 (31 weeks) | 10.7 | 377 | 134 | 4.0 | 2.01 | 31 | 1.17 | 0.77 |
| 18/01/23 (32 weeks) | 8.8 | 376 | 133 | 4.2 | 2.09 | 30 | 0.89 | 0.83 |
| 24/01/23 (33 weeks) | 12.7 | 370 | 133 | 4.1 | 2.02 | 31 | 0.99 | 0.85 |
| 30/01/23 (34 weeks) | 11.3 | 310 | 133 | 4.3 | 1.99 | 30 | 1.14 | 0.82 |
